# Supplementary material for: Across-subjects classification of stimulus modality from human MEG high frequency activity
Source: PLoS Comput Biol. 2018 Mar 12;14(3):e1005938. doi: 10.1371/journal.pcbi.1005938 (PMC5864083; doi:10.1371/journal.pcbi.1005938)
Supplement: S1 Appendix — Classifier accuracies obtained when training and testing the random forest model within instead of across subjects. The procedure adopted a split-half cross-validation scheme including both the fitting of the beamformer and the classification modelling. (PDF) [file pcbi.1005938.s001.pdf]

Supporting information

for: Across-subjects classification of stimulus modality from human MEG high frequency activity

Britta U. Westner, Sarang S. Dalal, Simon Hanslmayr, Tobias Staudigl

To compare the across-subjects classification approach to a within-subject classification scheme, the combination of beamformer source reconstruction with the random forest classification method was applied to the data of individual subjects. The cross-validation procedure was adapted to a split-half cross-validation, since a five-fold cross-validation approach did not leave enough data per fold to reliably estimate the covariance matrix to fit the beamformer.
The accuracies from the within-subject classifications were tested against chance level with a binomial tests, taking into account the indiviual trial numbers per subject. The level of significance was corrected for multiple comparisons using the Bonferroni method.

Eight out of the twenty subjects yield significant classification results, the averaged classification accuracy across those single-subject analyses is 60.31 % (Fig 1).
The drop in performance compared to the across-subjects model can be explained by the following factors: first, the model is fitted on a smaller sample of trials. While the across-subjects classifier is trained on the data of all subjects but one, the random forest in the individual subject model is trained on only half of a given subject's dataset. This also affects the accuracy of the beamformer source localization, as the covariance matrix is estimated on less sample points. Second, while the training of a within-subjects classifier in subjects with a rather noisy dataset might fail, this dataset is only a small fraction of the whole data contributing to the across-subjects classifier and thus has less impact. Furthermore, the across-subjects classifier can still be successfully applied to predict the classes from subjects with noisier datasets, as our analysis suggests.

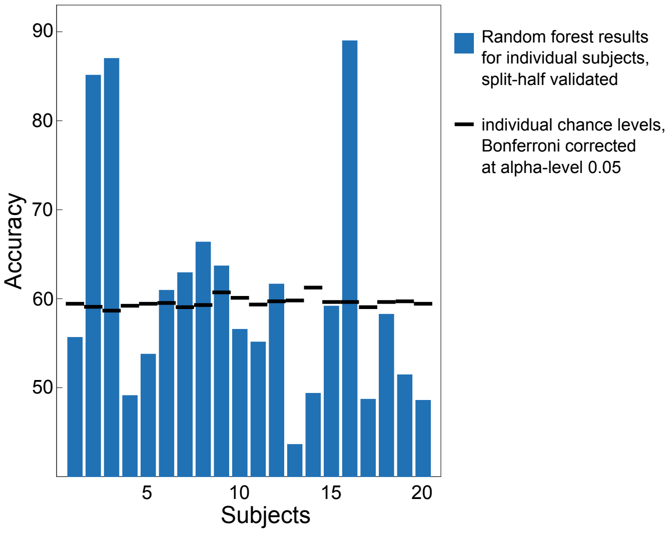

Fig 1. Classifier results for within-subject classification. Classifier accuracies obtained when training and testing the random forest model within instead of across subjects. The procedure adopted a split-half cross-validation scheme including both the fitting of the beamformer and the classification modelling.
